# Bone health post-stroke: a survey of stroke care physiatrists in Canada

**DOI:** 10.2340/jrm-cc.v8.43707

**Published:** 2025-09-03

**Authors:** Jamie L. FLEET, Nicole BILLIAS, Alexandria ROA AGUDELO, Ujjoyinee BARUA, Sydney KNIGHT, Robert TEASELL, Kristin K. CLEMENS

**Affiliations:** ^1^Department of Physical Medicine and Rehabilitation, Schulich School of Medicine and Dentistry, Western University; ^2^Lawson Research Institute; ^3^Department of Medicine, Division of Endocrinology, Western University, London, Canada

**Keywords:** stroke, osteoporosis, fractures, bone health, physiatry, survey

## Abstract

**Objective:**

People who have experienced stroke are at a high risk for falls, fractures, and osteoporosis. Bone health post-stroke is often overlooked. The goal of this study was to understand current practice perspectives and barriers to bone health care post-stroke among physiatrists.

**Methods:**

We conducted an online survey of English-speaking stroke physiatrists practicing in Canada from October 2023 to April 2024. We recruited participants through the Canadian Association of Physical Medicine and Rehabilitation newsletter and direct contact via hospital or university email. The survey included demographic and multiple-choice questions as well as open-ended queries. Data were summarized using frequencies and percentages, and open-ended questions were assessed qualitatively for themes.

**Results:**

Twenty-two physiatrists completed the survey. Female physiatrists made up 45.5% of respondents, and 36.4% were in their first 5 years of practice. Most worked in an academic hospital (81.8%). The majority (81.9%) of respondents felt there is a need for post-stroke bone health guidelines. Important themes that emerged from open-ended questions included a lack of awareness, research, and resources.

**Conclusions:**

In this study of Canadian physiatrists, most respondents feel post-stroke bone health guidelines would be beneficial. More research and resources focused upon bone health in this population is needed.

## LAY ABSTRACT

People who have had a stroke are at risk of falls. Changes also happen after the stroke that make bones more brittle which means a higher risk of breaking a bone during these falls. Physiatrists often follow patients after their stroke and may be able to provide guidance to prevent broken bones, however, no guidelines exist currently. This study surveyed stroke rehabilitation physiatrists in Canada to explore their thoughts and recommendations for bone health guidelines post stroke. The survey found that other priorities exist that make it difficult to manage bone health post stroke, but most respondents recommend developing specific bone health guidelines in the future.

People who have experienced stroke can have changes to their bone mineral density (BMD), especially in their hemiplegic limbs, even within the first year post stroke (1) leading to post-stroke osteoporosis (2). Other factors besides hemiplegia, such as advancing age, post-stroke inflammation, reduced sunlight exposure, and nutritional deficiencies also contribute (3). They also have a high risk of falls, with a previous Cochrane review reporting up to 73% of individuals having a fall within the first year after a stroke (4). Their high risk of falls, combined with lower BMD, can lead to fractures. A previous study showed individuals had a 5.7% risk of fracture during the 2 years post-stroke (5).

Despite their risk, individuals post-stroke are infrequently screened and treated for osteoporosis. Kapoor et al. assessed 16,581 patients post-stroke and found that only 5.1% underwent BMD testing (6). Part of the reason behind the lack of screening may be due to a lack of guidelines in this area. Current Canadian Stroke Best Practice Recommendations discuss falls prevention, but there are no specific recommendations for the screening or management of osteoporosis post-stroke (7). Similarly, Osteoporosis Canada guidelines do not consider stroke as a secondary cause of osteoporosis (8).

A previous survey of stroke physicians in the United Kingdom by Gaskell et al. found that over half underestimated the risk of fracture post-stroke and that despite considering falls risk, almost all respondents (89.5%) did not assess fracture risk (9). It may be that there are too many competing clinical considerations for post-stroke care and high mortality rates in the early post-stroke period for stroke physicians to focus on bone health (9). Physiatrists, however, play a critical role in stroke recovery, often following people longer than physicians on an acute stroke ward. The ideal time to optimize post-stroke bone health may be in the rehabilitation period. Understanding the current views of stroke physiatrists on the management of bone health post-stroke is a strategic first step before implementing guidelines for stroke rehabilitation.

Our goal was to explore awareness, current management strategies, and barriers to post-stroke bone health among Canadian physiatrists.

## METHODS

### Design, setting, and participants

We conducted a cross-sectional online survey of English-speaking physiatrists who provide stroke care in Canada. Physiatrists must have been practicing for at least 1 year and be able to complete the survey by study closure, 30 April 2024. Stroke did not need to make up the physiatrist’s entire practice, but the individual must provide at least some post-stroke care.

### Sampling and recruitment

Recruitment was done via convenience sampling. A link to the online survey was disseminated via the Canadian Association of Physical Medicine and Rehabilitation (CAPMR) newsletter. In addition, physiatrists were contacted directly via electronic correspondence through hospital or university affiliated email.

### Sample size

There are approximately 600 physiatrists in Canada, with around 100 of these practicing with individuals post-stroke. Based on a 95% confidence level and a 5% margin of error, we aimed for a sample size of 80.

### Survey

Our survey was developed using previously described methods for quantitative online surveys for health care professionals (10) and is reported based on the Standards for Reporting Qualitative Research (SRQR) (Appendix SI) (11). The study data were collected and managed using REDCap (Research Electronic Data Capture) (12, 13) through Lawson Health Research Institute. REDCap is a secure, web-based software platform designed to support data capture for research studies. The first page of the survey was a secure electronic consent form created through REDCap (14). Full survey is available in Appendix SII.

### Analysis

Quantitative survey and demographic data were summarized using proportions and percentages. Descriptive analyses of frequencies were completed using IBM SPSS Statistics 29.0.

Open-ended (qualitative) survey data were analyzed descriptively for themes. The research team developed thematic codes based on discussion of the open-ended survey data, then discussed and finalized these codes until a coding framework was agreed on, based on recommended practices of qualitative work (15, 16). The qualitative segments were then dual coded by a research assistant and associate and reviewed by the lead researcher to establish inter-rater reliability. The research team noticed themes, patterns, and unique findings in the coded segments of data, which was critically discussed among the team.

### Ethics

Our study was approved by Western University’s Research Ethics Board. Submission of the completed survey indicated implied consent. No personal identifiers were collected. All survey data were stored via REDCap through Lawson Health Research Institute. Data were only available to select study investigators.

## RESULTS

Twenty-two physiatrists completed our survey, of which 45.5% were female. The majority (81.8%) of respondents practiced in an academic hospital and in a large urban setting (86.4%). Demographic information is summarized in Table I.

**Table I:** Demographic information of 22 survey respondents

Characteristic	Number (%) *n* = 22
Age	
31–40 years	9 (40.9)
41–50 years	5 (22.7)
51–60 years	6 (27.3)
61–70 years	2 (9.1)
Gender	
Male	11 (50.0)
Female	10 (45.5)
Non-binary	1 (4.5)
Health Care Setting	
Academic Hospital	18 (81.8)
Community Hospital	3 (13.6)
Private practice	1 (4.5)
Community Setting	
Urban (population > 300,000)	19 (86.4)
Suburban (population 30,000–300,000)	3 (13.6)
Years of practice	
1–5	8 (36.4)
6–10	3 (13.6)
11–15	3 (13.6)
16–20	2 (9.1)
21–25	4 (18.2)
> 25	2 (9.1)
Proportion of practice focusing on patients post-stroke	
< 25%	6 (27.3)
25–50%	5 (22.7)
50–75%	10 (50.0)

Most respondents (86.4%) use the Canadian Stroke Best Practice Guidelines in their care of individuals post-stroke. The majority were also aware of common post-stroke complications such as depression (95.5%), fatigue (95.5%) and all were aware of spasticity, post-stroke pain, and cognitive impairment. Over 90% were aware of an increased risk of falls post-stroke and 59.1% reported familiarity with post-stroke osteoporosis. One respondent reported they were very familiar with bone health changes post-stroke, and 31.8% reported they were somewhat unfamiliar or very unfamiliar with these changes.

Most respondents were aware of Osteoporosis Canada’s Clinical Practice Guidelines for Diagnosis and Management of Osteoporosis (95.5%), and 57.1% of respondents felt that the osteoporosis guidelines were relevant to individuals post-stroke. Most (78.3%), however, felt there was a need for guidelines specifically for the post-stroke population. Important considerations for what to include in these guidelines included when to screen, lifestyle and exercise recommendations, as well as medical management options (Appendix SIII).

Two respondents reported that they routinely order BMD testing post-stroke, while 6 reported that they sometimes order BMD testing. Respondents stated that they were more likely to order a BMD in those with known osteoporosis, high falls risk, history of fracture, or other osteoporosis risk factors. Reasons for not ordering a BMD test are outlined in Table II.

**Table II:** Reasons preventing physiatrists from ordering a bone mineral density test

Reasons	*n* (%)
I am not sure when it is appropriate to order a BMD test	5 (22.7)
I do not think it is necessary	3 (13.6)
It is the responsibility of another healthcare professional who cares for these patients	9 (40.9)
Other*:	
No clear guideline for when to order post-stroke and not following long term	1 (4.5)
Unsure how I will interpret results	1 (4.5)

BMD: bone mineral density.

* Free text responses.

### Open-ended themes

Three questions in the survey were open-ended and were analyzed qualitatively for themes.

*Gaps in bone health screening and management post-stroke.* Lack of awareness, lack of guidelines and education, lack of evidence and research, competing symptoms and complications associated with the complexity of stroke, and lack of resources emerged as reasons for gaps in bone health screening and management of osteoporosis post-stroke. Themes with illustrative quotes are provided in Table III.

**Table III:** Themes and illustrative quotes to explain gaps in bone health screening post-stroke

Theme	Illustrative quotes
Lack of Awareness	“I am not sure if people are being screened or not … how many fractures occur in the context of stroke recovery.” “Bone health is not often considered as directly stroke related. I’m not aware of evidence that stroke itself physiologically impacts bone the way that [spinal cord injury] does…”
Lack of Guidelines	“…When there isn’t a clear guideline and there is a lot else medically going on, I think it gets forgotten…”
Lack of Evidence	“…Lack of evidence of [bone mineral density] in paretic limbs/post-stroke. Lack of evidence in different osteoporosis treatments post-stroke…” “…Data is lacking for this specific population…”
Complexity of Post Stroke Care	“…Some [physicians] may also feel that many high-risk individuals are not very mobile and may not be at as high a risk of injury, so they do not investigate (despite the fact that they have reduce mobility makes them higher risk of [osteoporosis] and fracture from a fall) …” “…With stroke, many other issues needing to be addressed in a time sensitive way (secondary prevention, follow-up with providers) …” “…Lack of clarity on difference in screening specific to stroke vs general population … Unique characteristics of people with stroke (spectrum is vast).”
Lack of Resources	“…‘Management fatigue’ – only so many issues that can be focused on at any given time…” “…Difficulty accessing [dual-energy x-ray absorptiometry scan] in smaller communities…” “…Rehab is poorly funded. We have no access to team supports…”
Lack of Follow-Up	“…I tend to see patients who are within the first 6–12 months post-stroke, so I don’t often have longer term follow up with them…” “…Limited availability of physiatrists to follow patients post hospital discharge…”
Ambiguous Responsibility	“…I think we usually leave bone health screening to the family doctors even after stroke…” “…probably just assumed they will get screened sooner or later in the regular guidelines…” “…Lack of clarity on who’s role it is to screen and manage…”

Lack of awareness included several key areas such as not knowing when to screen, not knowing about the risk of osteoporosis post-stroke or prevalence of fractures in this population, or not knowing how to interpret BMD results. The lack of awareness could stem from lack of guidelines and lack of research or evidence in this area, which were also highlighted as important potential gaps.

The multitude of issues that can arise in post-stroke care and how these things interact was also brought up as a potential contributor to lack of screening and management. Some found that bone health and osteoporosis screening are often not prioritized due to higher-priority concerns in post-stroke management, which may include things like dysphagia, pain, or secondary stroke prevention. Others mentioned the unique and diverse clinical presentations and characteristics of stroke and osteoporosis, which could make it difficult to manage bone health in this specific patient population.

Finally, respondents explained that lack of resources contributed to the gap in bone health screening and management post-stroke. Within this theme, resources mentioned were varied and included time, money, personnel/staff, as well as screening tools. Several important subthemes within the lack of resources main theme also emerged. These include specifically lack of long-term follow-up from physiatrists, as respondents found that they have limited ability to follow-up with patients’ post-hospital discharge, or on a long term basis, and that this is a factor that contributes to poor bone health screening and management post-stroke. In addition, the ambiguity surrounding whose role it is to screen for bone health issues post-stroke was discussed, with many respondents assuming that either general practitioners would screen for osteoporosis and follow-up with post-stroke patients, or that patients would eventually undergo osteoporosis screening via pathways in place for the general population.

*Improving screening and management of osteoporosis post-stroke.* When asked what would be helpful to improve screening and management of osteoporosis in post-stroke patients, respondents mentioned guidelines, education, and resources (i.e. technology, staff). Of all these strategies, guidelines emerged as the most prominent theme, with most respondents noticing that new evidence-based, stroke-specific guidelines would help improve screening and management of osteoporosis in patients post-stroke. Some respondents suggested specific screening guidelines (i.e. when to screen, how to screen, who should screen), or changes to current guidelines (i.e. add to the Heart and Stroke Foundation’s Post-Stroke Checklist, [originally developed by the Global Stroke Community Advisory Panel (17)], or incorporate recommendations for patients post-stroke within the current osteoporosis guidelines). Many respondents found that education would be a helpful strategy, via webinars, research articles, online accredited training, and more inclusion/introduction to the topic of stroke and bone health including medication and activity-based treatment exposure in residency training. Other suggestions included having a nurse to aid in screening and having an easy-to-use algorithm for risk stratification. Lastly, respondents suggested more medical research, better evidence to inform recommendations, and better knowledge translation tools.

*Future guideline development.* Respondents’ suggestions regarding who should be responsible for developing guidelines included: (*i*) physicians/primary healthcare providers, (*ii*) government-level (i.e. medical/healthcare system), and (*iii*) multi-disciplinary organizations/associations. Specific health care providers’ suggested and associated percentages are listed in Appendix SIV, however over half of respondents felt that physiatrists themselves should be involved in guideline development. Many respondents found that collaboration between specialities was important. Multidisciplinary organizations/associations were also mentioned by a handful of respondents as who should be responsible for developing guidelines for the management of bone health post-stroke. These included the Evidence-Based Review of Stroke Rehabilitation (EBRSR) (18), Canadian Stroke Best Practice Recommendations (7), and Osteoporosis Canada (8).

## DISCUSSION

Our study found that in a sample of physiatrists caring for individuals post-stroke, most do not adequately address bone health. This is primarily due to a lack of awareness, clear guidelines, resources, or a combination of all the above.

Our findings are in keeping with a previous UK survey focused on stroke physicians – not physiatrists – which found that a lack of awareness of the issue and lack of specific guidelines contributed to lack of screening (9). Participants in that study also found an issue with oversight of post-stroke bone health due to the many other competing medical issues seen post-stroke, which was also seen in our study. However, with the large impact on quality of life and mobility that can occur with osteoporosis and subsequent fractures (19, 20), we would argue that bone health should be an integral part of post-stroke care. Furthermore, physiatrists may be the optimal group to implement recommendations regarding bone health given the focus on quality of life and promotion of independence in the specialty. However, it is worth noting that despite physiatrists being the optimal group to manage bone health, this does not negate the multiple other stroke rehabilitation priorities and lack of resources that currently prevent adequate focus in this area. Integrating a bone health champion, similar to a fracture liaison specialist (21, 22), into inpatient stroke rehabilitation wards or outpatient clinics may help to minimize this burden.

Some respondents reported that they felt most individuals post-stroke would have screening eventually, based on their age. However, this assumption may not be true. Previous studies suggest that screening rates for osteoporosis are quite low even in the general population. For example, Gillespie et al. showed that only 26.5% of women between the ages of 65 and 79 years were screened for osteoporosis (23). Similarly, a study performed by Cheng and Green found that approximately 20% of men over the age of 65 had undergone osteoporosis screening (24). This is in contrast to a study done specifically on individuals post-stroke who showed only 5.1% of individuals underwent screening after their stroke (6).

There are limitations to this study. As with the majority of physician surveys (25), response rates were low, and we did not reach our target sample size. Furthermore, as in many surveys, this was subject to both recall and volunteer bias. Another limitation is that this study was restricted to physiatrists, yet there are many other important post-stroke stakeholders when considering bone health, including geriatricians, endocrinologists, rheumatologists, family physicians, nurse practitioners, and others. Future studies warrant a broader approach to gain further information from other healthcare providers.

Results from this survey have implications for future research directions as well as clinical practice. Physiatrists want more research on bone health post-stroke, including when to screen, and how to manage. Ideally, studies assessing changes in BMD or other bone parameters over time, starting from time of stroke into the chronic phase of recovery, could help guide when to screen. Large observational studies and/or randomized controlled trials on therapeutic options for management might also be helpful. Clinically, the survey also highlights the need for more widespread access to BMD scanning as well as algorithms or clinical decision tools for bone health post-stroke.

## CONCLUSIONS AND IMPLICATIONS

Most physiatrists in our survey suggest a need for specific guidelines for post-stroke osteoporosis. Future research to guide evidence-based treatment and prevention of osteoporosis post-stroke is also warranted.

## Supplementary Material


