# Supplementary material for: Bone health post-stroke: a survey of stroke care physiatrists in Canada
Source: J Rehabil Med Clin Commun. 2025 Sep 3;8:43707. doi: 10.2340/jrm-cc.v8.43707 (PMC12416335; doi:10.2340/jrm-cc.v8.43707)
Supplement: Supplementary file 1 [file JRMCC-8-43707-s1.pdf]

Supplementary material has been published as submitted. It has not been copyedited, or typeset by Journal of Rehabilitation Medicine – Clinical Communication

Appendix SI. Standards for Reporting Qualitative Research (SRQR) Checklist

		Reporting Item	Page Number
Title and Abstract			
Title	#1	Concise description of the nature and topic of the study identifying the study as qualitative or indicating the approach (e.g. ethnography, grounded theory) or data collection methods (e.g. interview, focus group) is recommended	Title page
Abstract	#2	Summary of the key elements of the study using the abstract format of the intended publication; typically includes background, purpose, methods, results and conclusions	1
Introduction			
Problem formulation	#3	Description and significance of the problem / phenomenon studied: review of relevant theory and empirical work; problem statement	3
Purpose or research question	#4	Purpose of the study and specific objectives or questions	3, 4
Methods			
Qualitative approach and research paradigm	#5	Qualitative approach and guiding theory if appropriate; identifying the research paradigm is also recommended; rationale. The rationale should briefly discuss the justification for choosing that theory, approach, method or technique rather than other options available; the assumptions and limitations implicit in those choices and how those choices influence study conclusions and transferability. As appropriate the rationale for several items might be discussed together.	4,5
Researcher characteristics and reflexivity	#6	Researchers' characteristics that may influence the research, including personal attributes, qualifications / experience, relationship with participants, assumptions and / or	N/A

		presuppositions; potential or actual interaction between researchers' characteristics and the research questions, approach, methods, results and / or transferability	
Context	#7	Setting / site and salient contextual factors; rationale	4
Sampling strategy	#8	How and why research participants, documents, or events were selected; criteria for deciding when no further sampling was necessary (e.g. sampling saturation); rationale	4
Ethical issues pertaining to human subjects	#9	Documentation of approval by an appropriate ethics review board and participant consent, or explanation for lack thereof; other confidentiality and data security issues	5
Data collection methods	#10	Types of data collected; details of data collection procedures including (as appropriate) start and stop dates of data collection and analysis, iterative process, triangulation of sources / methods, and modification of procedures in response to evolving study findings; rationale	4, Appendix A
Data collection instruments and technologies	#11	Description of instruments (e.g. interview guides, questionnaires) and devices (e.g. audio recorders) used for data collection; if / how the instruments(s) changed over the course of the study	Appendix A
Units of study	#12	Number and relevant characteristics of participants, documents, or events included in the study; level of participation (could be reported in results)	5, Table 1
Data processing	#13	Methods for processing data prior to and during analysis, including transcription, data entry, data management and security, verification of data integrity, data coding, and anonymisation / deidentification of excerpts	5
Data analysis	#14	Process by which inferences, themes, etc. were identified and developed, including the researchers involved in data analysis; usually references a specific paradigm or approach; rationale	5
Techniques to enhance trustworthiness	#15	Techniques to enhance trustworthiness and credibility of data analysis (e.g. member checking, audit trail, triangulation); rationale	N/A
Results/Findings			

Syntheses and interpretation	#16	Main findings (e.g. interpretations, inferences, and themes); might include development of a theory or model, or integration with prior research or theory	5-9,
Links to empirical data	#17	Evidence (e.g. quotes, field notes, text excerpts, photographs) to substantiate analytic findings	Table 4
Discussion			
Integration with prior work, implications, transferability and contribution(s) to the field	#18	Short summary of main findings; explanation of how findings and conclusions connect to, support, elaborate on, or challenge conclusions of earlier scholarship; discussion of scope of application / generalizability; identification of unique contributions(s) to scholarship in a discipline or field	9
Limitations	#19	Trustworthiness and limitations of findings	10
Other			
Conflicts of interest	#20	Potential sources of influence of perceived influence on study conduct and conclusions; how these were managed	Title page
Funding	#21	Sources of funding and other support; role of funders in data collection, interpretation and reporting – no funding	Title page

Appendix SII. Survey Items

1. What is your age?
   a. 20-30
   b. 31-40
   c. 41-50
   d. 51-60
   e. 61-70
   f. 71-80
   g. >80
   h. I prefer not to answer

2. What term best describes your gender?
   a. Male
   b. Female
   c. Non-binary
   d. Other – specify
   e. I prefer not to answer

3. How would you describe the health care setting that you work in?
   a. Academic hospital
   b. Community hospital
   c. Community clinic
   d. Other (specify)

4. How would you describe the community your practice is in?
   a. Urban (large city, population >300,000)
   b. Suburban (small city/town, population 30,000-300,000)
   c. Rural (outside of a town/city, population <30,000)

5. Number of years in practice?
   a. 1-5 years
   b. 6-10 years
   c. 11-15 years
   d. 16-20 years
   e. 21-25 years
   f. 26-30 years
   g. 30+ years

6. **Approximately** what proportion of your patient population is composed of patients whose **main condition is related to stroke**?
   a. <25%
   b. 25-50%
   c. 50-75%
   d. >75%

7. **In general**, do you utilize the **Canadian stroke best practice guidelines** when treating your stroke population?
   a. Yes
   b. No
   c. I don't know

7a. If b or c: Are you familiar with where to access the **Canadian stroke best practice guidelines**?

   a. Yes
   b. No

8. What are some common **post-stroke health issues** that you are familiar with (choose all that apply)?
    a. Osteoporosis/Fractures
    b. Falls
    c. Depression
    d. Fatigue
    e. Spasticity
    f. Pain
    g. Cognitive impairment
    h. I am not familiar with any of these health changes post-stroke (if selected, deselect response options a-g)

9. How familiar are you with **bone health changes** post-stroke?
    a. Very familiar
    b. Somewhat familiar
    c. Neutral
    d. Somewhat unfamiliar
    e. Very unfamiliar

10. Are you aware of any **guidelines for bone health screening** post-stroke?
    a. Yes
    b. No

If yes – please list guidelines -----

11. Are you familiar with Clinical Practice Guidelines for the Diagnosis and Management of Osteoporosis in Canada?
    a. Yes
    b. No

11a. If Yes: Do you feel the **Canadian Clinical Practice Guidelines** are relevant to individuals post-stroke?

a. Yes

b. No

c. I don't know

12. What screening procedures do you utilize to assess **fracture risk** in your stroke population (choose all that apply)?
    a. Bone mineral density (BMD)/DXA scan
    b. Clinical tools (FRAX, CAROC, etc)
    c. Laboratory testing (vitamin D, calcium)
    d. Other – specify
    e. I don't know
    f. I do not screen for fracture risk in my stroke population

13. Do you order a **bone mineral density test (BMD)** post-stroke?
    a. Yes
    b. No
    c. Sometimes – specify
    d. I don't know

13a. If b (no), what **prevents** you from **ordering BMD tests** (choose all that apply)?

a. I am not sure when it is appropriate to order a BMD test
b. I have other osteoporosis screening methods that I prefer
c. I do not think it is necessary
d. It is the responsibility of another health care professional who cares for these patients
e. Other: specify

14. Do you assess for the **risk of falls** post-stroke?

a. Yes
b. No
c. Sometimes – specify
d. I don't know

14a. if a (yes), what is **included** in your falls risk assessment (choose all that apply)?

a. Cognitive screening
b. Vision screening
c. Mobility and balance assessment
d. Sensation
e. Spasticity
f. History of falls
g. Medication review
h. Other – specify

14b. If b (no) what **prevents** you from **screening for falls** (choose all that apply)?

a. It is too time consuming
b. I am unsure what to ask
c. I do not think it is necessary
d. It is the responsibility of another health care professional seeing these patients
e. Other – specify

15. Do you feel that there is a **need for guidelines for bone health** post-stroke?

a. Yes
b. No
c. I don't know

15a if a (yes) what would be important to consider in these guidelines (choose all that apply)?

a. When to screen
b. Lifestyle modification – alcohol, smoking
c. Exercise
d. Fall prevention
e. High risk medication review
f. Medication management options
g. Other – specify
h. I don't know

15b. If b (no): Please specify why you feel there is no need for guidelines for bone health post-stroke (specify with free text)

16. Currently, there is a gap in bone health screening and management of osteoporosis in patients post-stroke

16a. Why do you think this is? (free text)
16b. What do you think would be helpful to improve screening and management in this area? (free text)
16c. Who should be responsible for developing guidelines for the management of bone health post-stroke? (free text)

Appendix SIII. Important considerations for future guidelines for bone health post-stroke

Reasons	n (%)
When to screen	18 (81.8)
Lifestyle modification*	14 (63.6)
Exercise recommendations	16 (72.7)
Fall prevention strategies	15 (68.2)
High risk medication review^	17 (77.3)
Medication management options	18 (81.8)

*Lifestyle modification included alcohol and smoking cessation, diet modification
^High risk medications could include, for example, corticosteroids, proton pump inhibitors, anticonvulsants, selective serotonin reuptake inhibitors, hormonal therapies(27)

Appendix SIV. Suggestions for Specific Health Care Providers to be Involved in Guideline Development

Health Care Provider	Number (%) *
Physiatrists	12 (54.5)
Endocrinologists	8 (36.4)
Neurologists	4 (18.2)
Family physicians/General practitioners	4 (18.2)
Rheumatologists	2 (9.1)
Geriatricians	2 (9.1)
Physiotherapists	2 (9.1)
Internists	1 (4.5)
Occupational therapists	1 (4.5)

*Respondents were able to list more than one health care provider, while others listed no health care providers in this section, meaning that the total percentage does not equal 100%
